# High Occurrence of Thrombo-Embolic Complications During Long-Term Follow-up After Pleural Infections—A Single-Center Experience with 536 Consecutive Patients Over 17 Years

**DOI:** 10.1007/s00408-020-00374-x

**Published:** 2020-06-30

**Authors:** Henna Maria Ala-Seppälä, Mika Tapani Ukkonen, Antti Ilmari Lehtomäki, Emilia Susanna Pohja, Jaakko Juhani Nieminen, Jari Olavi Laurikka, Jahangir Ari Khan

**Affiliations:** 1 Department of Cardio-Thoracic Surgery, Tays Heart Hospital, Tampere University Hospital, Ensitie 4, 33520 Tampere, Finland; 2 Department of Gastroenterology and Alimentary Tract Surgery, Tampere University Hospital, P.O. Box 2000, 33521 Tampere, Finland; 3 Faculty of Medicine and Life Sciences, Tampere University, Arvo Ylpön katu 34, 33520 Tampere, Finland

**Keywords:** Deep vein thrombosis, Empyema, Pleural infection, Pulmonary embolism, Venous thrombo-embolism

## Abstract

**Purpose:**

Pleural infections are associated with significant inflammation, long hospitalizations, frequent comorbidities, and are often treated operatively—all of which are consequential risk factors for thrombo-embolic complications. However, their occurrence following the treatment of pleural infection is still unknown. The aim of the study was to ascertain the early and long-term occurrence of thrombo-embolic events in patients treated for pleural infections.

**Methods:**

The study included all patients that were treated for pleural infections in Tampere University Hospital between January 2000 and December 2016. Data regarding later treatment episodes due to pulmonary embolisms and/or deep vein thromboses as well as survival data were requested from national registries. The rates were also compared to a demographically matched reference population adjusted for age, sex, and the location of residence.

**Results:**

The final study population comprised 536 patients and 5318 controls (median age 60, 78% men). The most common etiology for pleural infection was pneumonia (73%) and 85% underwent surgical treatment for pleural infection. The occurrence of thrombo-embolic complications in patients and controls was 3.8% vs 0.1% at three months, 5.0% vs 0.4% at one year, 8.8% vs 1.0% at three years, and 12.4% vs 1.8% at five years, respectively, *p* < 0.001 each. Female sex, advanced age, chronic lung disease, immunosuppression, video-assisted surgery, and non-pneumonic etiology were associated with a higher incidence of thrombo-embolism.

**Conclusions:**

The occurrence of thrombo-embolic events—particularly pulmonary embolism but also deep vein thrombosis—was significant in patients treated for pleural infections, both initially and during long-term follow-up.

## Introduction

The occurrence of thrombo-embolic complications—including deep vein thrombosis and pulmonary embolism—following the treatment for pleural infections is unknown. A significant proportion of patients undergo operative treatment, either traditional open surgery or video-assisted thoracic surgery (VATS) [1, 2]. Surgery in general is one of the most important risk factors for thrombo-embolism [3–5]. In other non-cardiac chest surgery, thrombo-embolic complication rates of 0.2–12.1% have been described [6–10]. Pleural infections, furthermore, are associated with significant inflammation, long hospitalizations, immobilization, as well as other comorbidities, all of which are consequential risk factors [4, 11, 12].

The aim of the study was to ascertain the occurrence of thrombo-embolic complications following the treatment of pleural infections, both in patients that underwent surgical treatment and in patients that were treated conservatively. To further illustrate the degree of disease burden in these patients, the venous thrombo-embolic rates were also compared to that of a demographically matched reference population.

## Material and Methods

### Study Patients and Reference Population

The study was performed according to the Helsinki declaration. Institutional review board approval was obtained. The study included each consecutive patient that was treated for pleural infections in the Tampere University hospital, Tampere, Finland, a tertiary academic referral center, between January 2000 and December 2016. The patients were identified from the institutional database by retrieving all cases associated with the International Statistical Classification of Diseases and Related Health Problems (10th revision; ICD-10), diagnosis code “J86” or any subclass of “J86”. All subjects were carefully reviewed and excluded if there was no definitive evidence for the diagnosis of pleural infection. Furthermore, patients without adequate follow-up data and child patients treated in the pediatric department were excluded. The demographic information and relevant medical history were collected from each patient. Recovery data, including the length of hospitalization, the occurrence of complications, and/or the need for reoperations, as well as the results of relevant laboratory examinations, were also recorded. The demographically matched reference population was obtained by requesting a random sample of 10 individual controls matched for age, sex, and the location of residence for each patient from the Finnish Population Register Centre, a national registry that also provides large control materials for research purposes, and from which survival data including all deaths occurring until the end of November 2018 were obtained for each patient and control.

### The Diagnosis and Classification of Pleural Infections

The diagnosis of pleural infection required clinical signs of infection including fever and leukocytosis together with either a collection of pus in the pleural space or an effusion with other laboratory findings indicating infection, i.e., a positive bacterial culture and/or stain or polymerase chain reaction test, pH < 7.2, lactate dehydrogenase level > 1000 IU/L, and/or glucose < 2.2 mmol/L. The etiology of pleural infection was classified into the following groups: pulmonary infection, trauma, malignancy-related cases, procedural complication, and other or unknown etiology. Malignancy-related cases comprised spontaneous pleural infections occurring in patients with intra-thoracic malignancies. The patients were treated according to clinic standards and the course of treatment was categorized into non-surgical, with or without pleural drainage, or surgical. Surgery was either traditional open surgery or VATS.

### Thrombo-Embolic Complications

The clinic practice for prophylactic anticoagulation over the study period was subcutaneous injections of enoxaparin 40 mg daily during the hospitalization in both surgically and non-surgically treated patients. Anticoagulation was discontinued on hospital discharge, though when considered necessary, omitting from anticoagulation, for example due to bleeding, other doses, and/or extended prophylaxis, i.e., that continuing after discharge, as well as reinstitution of possible oral anticoagulants were subject to the discretion of the clinician. The occurrence of deep vein thrombosis or pulmonary embolism was obtained from the National institute for health and welfare database that contains complete data on all specialized medical care hospitalizations as well as emergency room and outpatient clinic visits in Finland that had occurred or begun before the 31 December 2016. The treatment episodes associated with ICD-10 diagnosis codes “I80”, “I82”, or “I26”, or any subclass of these diagnosis codes following the first treatment day for pleural infections in patients and the corresponding index date in controls were identified from the database. The incidence, type, and timing of thrombo-embolic complications were compared between patients and the reference population and the associations with patient subgroups and patient-specific risk factors were ascertained. The total number of hospital periods and in-hospital days related to venous thrombo-embolism was recorded. If a study subject had several treatment episodes for thrombo-embolism, only the first one was included in the incidence analysis as the approach could not differentiate whether later episodes were associated with the same or another thrombo-embolic episode. All episodes were included when analyzing the cumulative number of in-hospital days.

### Statistical Analysis

Statistical analyses were carried out using IBM SPSS Statistics version 25 statistical software for Windows (IBM corp. Armonk, NY, USA). The cumulative occurrence of venous thrombo-embolism was compared between patients and the reference population at several time points. All study subjects that did not complete the entire follow-up due to insufficient follow-up time or death during the follow-up were excluded from the corresponding analyses at each time point unless venous thrombo-embolic event had already occurred. Categorical variables were compared using the Chi-square and Fisher’s exact tests. Non-parametric variables were compared using the Mann–Whitney U test. Kaplan–Meier curves and the Log rank test were used to illustrate and compare the short- and long-term occurrence of venous thrombo-embolic events in patients and the reference population and included the cases with at least five years of follow-up, death within five years, and/or a venous thrombo-embolic event within five years. Independent risk factors for venous thrombo-embolism in patients were further ascertained using multivariable Cox regression analysis by including the variables with statistically significant associations in the univariable analyses. Statistical significance was set at *p* < 0.05.

## Results

Altogether 30 patients that were treated during the study period were excluded from the analysis due to unavailable follow-up data or matching controls, restrictions regarding registry use, recurrent disease, or foreign origin as information regarding later treatment episodes was not available from national registries for foreigners. For eight patients less than 10 demographically matching controls were available and they were included in the analysis. The final study population comprised 536 patients and 5318 demographically matched controls. The median follow-up time for patients and controls was 63 (interquartile range 21–105) months and the total cumulative number of person-years followed up was 2520 in patients and 26,488 in controls, respectively. The demographic information of the study population, the medical history of patients, the etiology of the pleural infection, as well as the type of treatment given are shown in Table 1. The most common cause for the pleural infection was pneumonia accounting for 73% of all cases and the majority of patients, 85%, underwent surgical treatment. Of surgically treated patients, 65% underwent open surgery and 35% VATS. The procedures entailed simple canalization in 28%, canalization and decortication in 38%, and extended procedures, i.e., lung or thoracic wall resections, pleurectomies, and/or fenestrations, in 34% of cases. The length of the hospitalization was similar between patients that underwent open surgery and VATS, 14 vs. 15 days, respectively, *p* = 0.593.

**Table 1:** The demographic information of patients and controls as well as the medical history and the etiology and treatment of pleural infections in patients

	Patients	Controls
N	536	5318
Median age (years)	60	60
Male sex	78%	78%
Diabetes	16%	
Coronary disease	13%	
Heart failure/LVEF^1^ < 50%	6%	
Hypertension	33%	
Dyslipidemia	15%	
Chronic lung disease	17%	
Smoking	38%	
Alcoholism	19%	
Immunosuppression	10%	
Etiology for pleural infection		
Pneumonia	73%	
Trauma	5.1%	
Malignancy	3.9%	
Iatrogenic	6.2%	
Other	11%	
Treatment		
Non-surgical^2^	15%	
Surgical	85%	

^1^Left ventricular ejection fraction

^2^With or without pleural drainage

The controls were obtained from national registries with a 1:10 ratio and were demographically matched for age, sex, and the location of residence

**Fig. 1:**
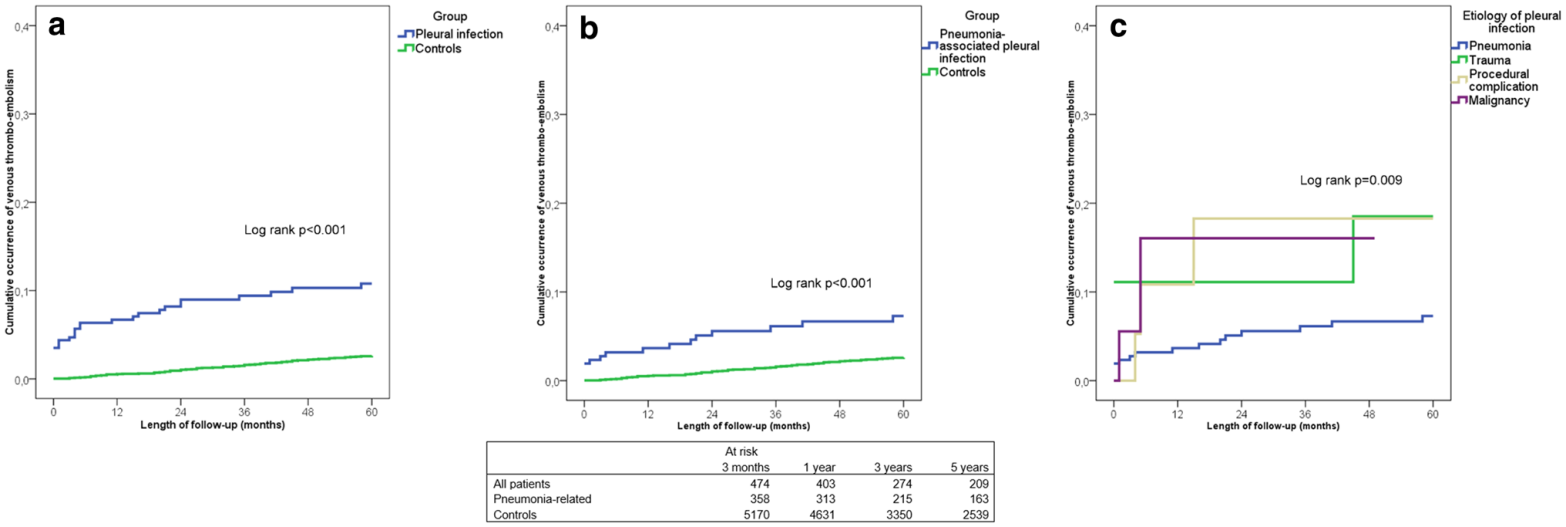
The cumulative occurrence of thrombo-embolic events in **a** all patients treated for pleural infections, **b** patients with pneumonia-related pleural infection, and matched controls, and **c** the occurrence of thrombo-embolic complications according to the etiology of pleural infection during five-year follow-up

The main results of the study are shown in Table 2 and Fig. 1. Both initially and during long-term follow-up, the occurrence of thrombo-embolic events—particularly pulmonary embolism but also deep vein thrombosis—was high in patients with pleural infections and manifold compared to that of the reference population. The mortality rates of patients and the reference population during follow-up are shown in Table 2 and the number of in-hospital days related to treatment periods associated with thrombo-embolic events in patients and the reference population is shown in Table 3. In the patient subgroup analyses, female sex, advanced age, chronic lung disease, immunosuppression, non-pneumonic etiology for the pleural infection, and VATS were associated with a higher occurrence of thrombo-embolic events in univariable analyses (Table 4). In multivariable analysis, non-pneumonic etiology and VATS were independently associated with a higher incidence of venous thrombo-embolism (Table 5). Smoking was associated with a lower risk for venous thrombo-embolism, though these patients were also significantly younger than non-smokers (median age 55 vs. 64, *p* < 0.001). The five-year mortality rate of reference subjects with venous thrombo-embolism was significantly higher than in those without, 42% vs. 9.1%, *p* < 0.001, respectively, but there were no differences in the mortality rates between patients with pleural infections who developed thrombo-embolic events and those that did not during five-year follow-up, 31% vs. 31%, respectively, *p* = 0.958.

**Table 2:** The cumulative proportions of all patients treated for pleural infections, patients with pneumonia-associated pleural infection, and matched controls with treatment episodes for venous thrombo-embolism during follow-up

	All patients	p-value	Pneumonia-associated pleural infection	p-value	Controls
Any venous thrombo-embolism					
3 months	3.8%	< 0.001	1.7%	< 0.001	0.1%
1 year	5.0%	< 0.001	1.9%	< 0.001	0.4%
3 years	8.8%	< 0.001	4.2%	< 0.001	1.0%
5 years	12.4%	< 0.001	6.7%	< 0.001	1.8%
Pulmonary embolism					
3 months	3.0%	< 0.001	1.4%	< 0.001	0.1%
1 year	3.7%	< 0.001	1.6%	< 0.001	0.3%
3 years	6.9%	< 0.001	3.7%	< 0.001	0.6%
5 years	10.0%	< 0.001	6.1%	< 0.001	1.2%
Deep vein thrombosis					
3 months	1.1%	< 0.001	0.6%	0.037	0.1%
1 year	1.7%	< 0.001	0.6%	0.151	0.2%
3 years	2.9%	< 0.001	1.4%	0.079	0.4%
5 years	3.8%	< 0.001	1.8%	0.128	0.7%
Mortality					
3 months	10.3%	< 0.001	7.1%	< 0.001	0.3%
1 year	17.7%	< 0.001	12.2%	< 0.001	1.9%
3 years	26.7%	< 0.001	20.4%	< 0.001	5.9%
5 years	31.3%	< 0.001	25.3%	< 0.001	9.0%

Statistical testing was performed by comparing the occurrence of thrombo-embolic events of the respective patient group to that of the controls at each time point. The study subjects that did not complete follow-up were excluded from the corresponding analyses at each time point

**Table 3:** The mean number of in-hospital days related to venous thrombo-embolism in patients treated for pleural infections and matched controls

	Patients In-hospital days (mean)	Controls In-hospital days (mean)	*p*
Any venous thrombo-embolism			
3 months	0.74	0.01	< 0.001
1 year	1.22	0.15	< 0.001
3 years	2.19	0.24	< 0.001
5 years	3.16	0.39	< 0.001
Pulmonary embolism			
3 months	0.57	0.01	< 0.001
1 year	0.76	0.11	< 0.001
3 years	1.33	0.17	< 0.001
5 years	2.03	0.28	< 0.001
Deep vein thrombosis			
3 months	0.25	0.01	< 0.001
1 year	0.56	0.04	< 0.001
3 years	1.00	0.07	< 0.001
5 years	1.32	0.11	< 0.001

Patients and controls not completing the entire follow-up were excluded from the corresponding analysis at each time point

**Table 4:** The univariable associations of patient characteristics with the occurrence of venous thrombo-embolism during one- and five-year follow-up

The occurrence of venous thrombo-embolism						
	One-year follow-up			Five-year follow-up		
	Number of events per patients at risk	%	p-value	Number of events per patients at risk	%	p-value
All patients	20/404	5%		26/209	12.4%	
Male sex	**11/316**	**3.5%**	**0.010**	17/166	10.3%	0.058
Female sex	**9/88**	**10.2%**	**0.010**	9/43	20.9%	0.058
Age ≥ 60 years	11/184	6.0%	0.384	**16/72**	**22.2%**	**0.002**
Diabetes	3/62	4.8%	> 0.999	3/24	12.5%	0.992
Coronary disease	1/44	2.3%	0.711	2/12	14.3%	0.688
Heart failure/LVEF^1^ < 50%	0/10	0%	> 0.999	0/2	0%	> 0.999
Hypertension	7/123	5.7%	0.650	8/48	16.7%	0.312
Dyslipidemia	2/46	4.3%	> 0.999	3/19	15.8%	0.713
Chronic lung disease	2/65	3.1%	0.754	**7/24**	**29.2%**	**0.008**
Smoking	**3/162**	**1.9%**	**0.019**	7/85	8.2%	0.127
Alcoholism	3/73	4.1%	> 0.999	4/34	11.8%	> 0.999
Immunosuppression	3/34	8.8%	0.232	**4/12**	**33.3%**	**0.047**
Etiology for pleural infection						
Pneumonia	**6/314**	**1.9%**	** < 0.001**	**11/163**	**6.7%**	** < 0.001**
Non-pneumonic etiology	**14/90**	**15.6%**	** < 0.001**	**15/46**	**32.6%**	** < 0.001**
Trauma	3/23	13.0%	0.097	3/14	21.4%	0.391
Malignancy	**2/6**	**33.3%**	**0.031**	**2/2**	**100%**	**0.015**
Iatrogenic	2/21	9.5%	0.279	2/9	22.2%	0.311
Other	**7/40**	**17.5%**	** < 0.001**	**8/21**	**38.1%**	** < 0.001**
Treatment						
Non-surgical^2^	3/55	5.5%	0.744	4/31	12.9%	> 0.999
Surgical	17/349	4.9%	0.744	22/178	12.4%	> 0.999
Open surgery	10/240	4.2%	0.380	**12/156**	**7.7%**	** < 0.001**
Video-assisted thoracic surgery	7/110	6.4%	0.423	**10/23**	**43.5%**	** < 0.001**

^1^Left ventricular ejection fraction

^2^With or without pleural drainage

Statistical comparisons were made between opposing groups, for example, between males and females

The study subjects that did not complete follow-up were excluded from the corresponding analyses. Statistically significant associations are highlighted in bold

**Table 5:** Multivariable Cox regression analysis for patient-specific risk factors for venous thrombo-embolism during five-year follow-up

	Hazard ratio	95% Confidence interval	p-value
Male sex	0.53	0.21–1.33	0.177
Age ≥ 60 years	1.58	0.65–3.81	0.309
Chronic lung disease	1.33	0.50–3.55	0.569
Smoking	0.65	0.24–1.72	0.387
Immunosuppression	1.84	0.58–5.87	0.301
Non-pneumonic etiology for pleural infection	2.99	1.29–6.97	0.011
Video-assisted thoracic surgery	4.57	1.82–11.46	0.001

Variables with statistically significant associations with the occurrence of venous thrombo-embolism in the univariable analyses were included in the model

## Discussion

Despite the relatively high frequency of pleural infections, only little is known about the prevalence of venous thrombo-embolism in these patients. The present study sought to describe their occurrence and consequently demonstrated that the occurrence of thrombo-embolic events—pulmonary embolism as well as deep vein thrombosis—was high and, during long-term follow-up, significantly greater when compared to a large demographically matched reference population.

Pneumonia, which is also the most frequent cause for pleural infections, has been associated with a substantial risk for venous thrombo-embolism [13–15]. In the present study, patients with pneumonia-related pleural infections clearly had a high occurrence of thrombo-embolic events, though they were even more prevalent in other patient subgroups, for example in those with cancer-associated pleural infections. Consistent with the current literature, advanced age and chronic lung disease were found to be associated with an increased risk for venous thrombo-embolism [10, 11, 16–18]. Pleural infections may be associated with pulmonary atelectasis and external lung compression in cases with extensive effusions, which may directly contribute to the risk of pulmonary embolisms in this cohort of patients. Furthermore, immobilization may be a particularly consequential risk factor during infections [19]. The association of venous thrombo-embolism with immunosuppression has been described earlier as well [4]. Smoking did not appear to be associated with a higher risk of venous thrombo-embolism in this study, probably because actively smoking patients were younger than non-smokers.

Most patients in the present series underwent surgery. In contemporary studies describing the incidence of venous thrombo-embolism following general thoracic surgery for other indication, mostly lung cancer, significantly lower (0.005–1.7%) rates have been reported, though higher percentages have also been observed in older series and when no perioperative antithrombotic prophylaxis was administered [6, 7, 20]. Despite routine in-hospital thrombo-prophylaxis, the early occurrence of thrombo-embolic events was relatively high in this study. Current guidelines do not clearly recommend thrombo-prophylaxis extending beyond the hospitalization period in patients treated for pleural infections. The results of the present study may suggest that at least some cases, particularly older patients, and those with non-pneumonic etiology for the disease, might benefit from a longer prophylaxis. As the long-term risk for thrombo-embolic events appears to be significantly elevated, close follow-up seems reasonable as well. It does not appear plausible that pleural infections or their treatments directly cause venous thrombo-embolism beyond the initial period and the later thrombo-embolic episodes are probably related to the high rates of comorbidities in these patients.

Somewhat surprisingly and in contrast to the controls, the occurrence of venous thrombo-embolism did not clearly impact the overall mortality rate of patients with pleural infections. It is possible that, as the overall morbidity and mortality rates of pleural infection patients were high, the development of venous thrombo-embolism did not as clearly identify the most high-risk cases as in unselected cohorts. There were no clear differences in the thrombo-embolic event rates between patients undergoing surgical and non-surgical treatment, though VATS appeared to be associated with a higher risk. In addition to limited statistical power in the subgroup analyses, the authors speculate that this may be due to patient selection, as the minimally invasive approach may have been more frequently selected for the most morbid cases, particularly in the early years of the series, and these cases represented those that were included in the long-term analyses. The overall length of hospitalization—and presumably the duration of thrombo-prophylaxis—did not differ between patients that underwent open surgery or VATS in this series.

There are several limitations regarding the study. The material consists of patients treated at a single University Hospital and may not accurately reflect the patient material at other institutions. Our results are more representative of patients undergoing surgical treatment as most patients in the series underwent surgical treatment for pleural infection. The diagnostics and treatment of both pleural infections and venous thrombo-embolism have evolved during the study period. Some selection bias is likely, as patients successfully treated conservatively and those with prohibitive risks regarding surgery were probably not referred. The retrospective setting of this study also created some limitations regarding the availability of some of the patient’s medical history data, which were also not available for the reference population, limiting subgroup analyses. While the exact prevalence of comorbidities in the control subjects was not known, they should, due to their large number, relatively accurately correspond to the standard population with comparable age- and sex-distributions and average prevalence of comorbidities. By including the reference population, our aim was to allow comparisons with the presumably standard rate of venous thrombo-embolism, particularly during long-term follow-up. Some patients may have been missed due to inaccurate use of diagnosis codes. The routine thrombo-prophylaxis protocol may not have been used in all patients and the proportion of patients later using oral anticoagulants was not known. The rate of deep vein thrombosis was relatively low in the patients, possibly because when concomitant deep vein thrombosis and pulmonary embolism were present only the diagnosis code of pulmonary embolism may have been used, and because some deep vein thromboses may have been diagnosed and treated in primary health care. However, most patients with clinically relevant acute symptomatic conditions, particularly pulmonary embolisms which are the most consequential thrombo-embolic complication, were included, even if they were treated at other hospital. The strengths of this study include a relatively large cohort of consecutive patients that were treated over a time period of nearly two decades, long follow-up time, and comprehensive national registries.

In conclusion, we report that the occurrence of thrombo-embolic complications, particularly pulmonary embolism, is significant in patients treated for pleural infections both initially and during long-term follow-up. Likewise, the overall mortality rate was high. Venous thrombo-embolism appears most frequent in females and in patients with advanced age, chronic pulmonary disease, immunosuppression, and non-pneumonic etiology for the pleural infection.

## Data Availability

Data not available.
